# Rhabdomyolysis Secondary to Severe Hypothyroidism Due to Hashimoto’s Thyroiditis: A Case Report

**DOI:** 10.7759/cureus.39919

**Published:** 2023-06-03

**Authors:** Salina Munankami, Manish Shrestha, Shefali Amin, Abhishek Bajracharya, Rubina Paudel

**Affiliations:** 1 General Medicine, Kathmandu Medical College, Kathmandu, NPL; 2 Internal Medicine, Tower Health Medical Group, Reading, USA; 3 Internal Medicine, Reading Hospital/Tower Health, West Reading, USA; 4 General Practice, Nepal Medical College, Kathmandu, NPL; 5 Internal Medicine, Reading Tower Health, Reading, USA

**Keywords:** hypothyroidism, thyroid-stimulating hormone (tsh), anti-thyroid peroxidase antibodies, creatine kinase, rhabdomyolysis, hashimoto’s thyroiditis

## Abstract

Hashimoto’s thyroiditis, a chronic autoimmune inflammation of the thyroid glands, is the most common cause of hypothyroidism in iodine-sufficient areas, which can have varied clinical manifestations. It is more common in females and usually has an insidious course. Most patients present with mild clinical symptoms, such as constipation, fatigue, and weakness. Symptoms are associated with a slight increase in thyroid-stimulating hormone (TSH) levels and the presence of thyroid antibodies. However, overt hypothyroidism is uncommon. We hereby present an interesting case of rhabdomyolysis secondary to severe hypothyroidism due to Hashimoto’s thyroiditis.

## Introduction

Hashimoto’s thyroiditis is the most common cause of hypothyroidism in iodine-sufficient regions worldwide. It is a chronic autoimmune inflammation of the thyroid tissue [1]. It is five to eight times more common in females compared to males [2]. Clinical symptoms are related to slow metabolic functions such as fatigue and weakness, constipation, and weight gain [3]. The usual course of the disease is a gradual loss of thyroid function, causing patients to have mild hypothyroidism. However, overt hypothyroidism can ensue in these patients at a rate of 5% per year, with patients presenting with features of severe hypothyroidism such as myxedema coma, impaired respiratory and cardiovascular function, or rhabdomyolysis [2,4]. We present an interesting case of overt hypothyroidism secondary to Hashimoto’s thyroiditis presenting as rhabdomyolysis.

## Case presentation

A 24‐year‐old male with no past medical history presented with generalized body aches and weakness for two weeks. The patient reported that the body aches were insidious in onset, progressive, and aggravated with activities of daily living with no significant relieving factors. These symptoms were also associated with constipation. The patient reported gaining 30 lb in the past six months without any changes in diet or exercise regimens. The patient denied fever, headache, hoarse voice, cold intolerance, hair loss, dysphagia, weight gain, and dizziness. He denied recent trauma, strenuous exercise, alcohol consumption, or medication use. He denied a family history of autoimmune diseases or thyroid disorders.

In the emergency department, he was bradycardic with a pulse rate of 57/minute (regular), non-tachypneic with a respiratory rate of 19/minute, hypertensive with a blood pressure of 145/79 mmHg, and afebrile with a temperature of 97.7°F. Physical examination was negative for thyroid enlargement, leg edema, eye changes, muscle wasting, hypertrophy, or focal weakness. Other system examinations were unremarkable.

Laboratory analysis (Table 1) was suggestive of severe hypothyroidism with thyroid-stimulating hormone (TSH) elevated at 200.3 uIU/mL (normal range: 0.4-5.3 uIU/mL), free T4 of <0.25 ng/dL (normal range: 0.58-1.64 ng/dL), and free T3 elevated at 4.92 ng/dL (normal range: 2.2-4.1 ng/dL). Creatine kinase (CK) was elevated at 1364 IU/L (normal range: 30-223 IU/L), suggesting rhabdomyolysis. The thyroid peroxidase (TPO) antibody was >900 IU/mL (normal range: <9 IU/L), and the thyroglobulin antibody was elevated at 23 IU/mL (normal range: <1 IU/mL), suggestive of Hashimoto’s thyroiditis.

**Table 1:** Laboratory analysis results on admission. HDL, high-density lipoprotein; LDL, low-density lipoprotein; TSH, thyroid-stimulating hormone

Laboratory Variables	Laboratory Values	Reference Range
Sodium (mmol/L)	138	136-145
Potassium (mmol/L)	3.5	3.5-5.1
Chloride (mmol/L)	102	98-107
CO_2_ (mmol/L)	23.2	21-31
Glucose (mg/dL)	108	70-99
Blood urea nitrogen (mg/dL)	13	7-25
Creatinine (mg/dL)	1.37	0.6-1.3
Calcium (mg/dL)	10.1	8.6-10.3
Anion gap (mmol/L)	13	5-12
Magnesium (mg/dL)	1.8	1.9-2.7
Albumin (g/dL)	5.1	3.5-5.7
Total protein (g/dL)	8.4	6.4-8.9
Lipase (IU/L)	38	11-82
Alkaline phosphatase (IU/L)	57	34-104
Aspartate aminotransferase (IU/L)	63	13-39
Alanine aminotransferase (IU/L)	37	7-52
Direct bilirubin (mg/dL)	0.1	0.0-0.2
Total bilirubin (mg/dL)	0.8	0.3-1.0
White blood cell count (×10^3^/µl)	9.8	4.8-10.8
Red blood cell count (×10^6^/µl)	4.98	4.5-6.1
Hemoglobin (g/dL)	14.8	14-17.5
Hematocrit (%)	41.8	39-53
Platelet count (×10^3^/µl)	289	130-400
Total cholesterol (mg/dL)	255	<200
Triglycerides (mg/dL)	177	<175
HDL cholesterol (mg/dL)	44	23-92
LDL calculated (mg/dL)	175.6	<100 optimal
Troponin (ng/mL)	<0.03	<0.06
Serum cortisol, morning (mcg/dL)	8.4	6.7-22.6
Creatinine kinase (IU/L)	1364	30-223
TSH (uIU/mL)	200.332	0.45-5.33
Free T4 (ng/dL)	<0.25	0.58-1.64
Free T3 (ng/dL)	4.92	2.2-4.1
Anti-thyroid peroxidase antibody (IU/mL)	>900	<9
Anti-thyroglobulin antibody (IU/mL)	23	<1

**Figure 1:**
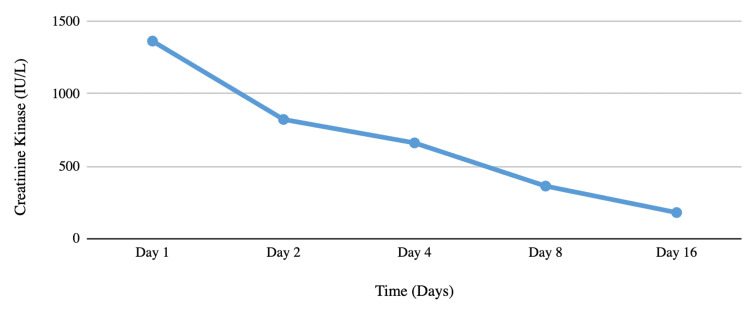
CK trend over time.

**Figure 2:**
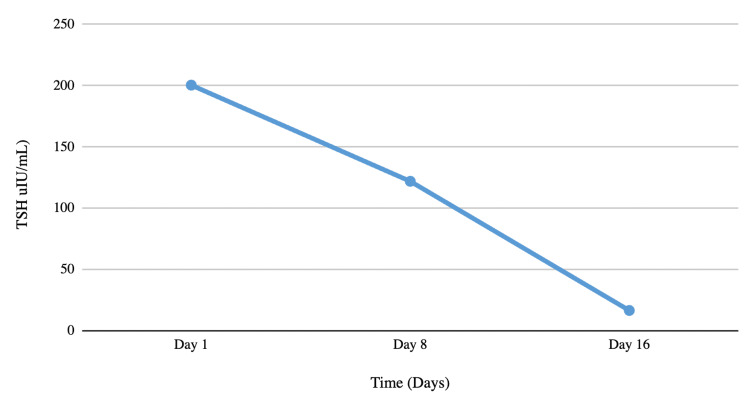
TSH trend over time. TSH: thyroid-stimulating hormone

The urine drug screen was negative. Electrocardiogram (EKG) and chest X‐ray were unremarkable. Based on the clinical and laboratory analysis, the patient was diagnosed with severe hypothyroidism secondary to Hashimoto’s thyroiditis with rhabdomyolysis. The patient was started on intravenous fluids and was started on oral levothyroxine 150 mcg orally daily. His symptoms improved with the treatment, and the levels of CK (Figure 1) and TSH also improved (Figure 2). The patient was discharged with a plan for outpatient follow-up.

## Discussion

Hashimoto’s thyroiditis is a chronic autoimmune thyroiditis characterized by gradual thyroid failure, with or without goiter formation, due to lymphocytic infiltrators and the autoimmune-medicated destruction of the thyroid tissue [1]. The pathophysiology consists of genetic susceptibilities such as familial clustering, association with human leukocyte antigen (HLA) alleles (DR3), and the linkage of immune-related genes with precipitating factors such as female sex, pregnancy, excessive iodine intake, and radiation exposure [5,6].

The clinical manifestations are secondary to a lack of thyroid hormones. This can lead to a generalized slowing of the metabolic process, causing slow movement and speech, cold intolerance, constipation, weight gain, bradycardia, and generalized fatigue [1]. Furthermore, the accumulation of matrix glycosaminoglycans in the interstitial spaces of many tissues causes puffy faces, enlargement of the tongue, and hoarseness [7]. Even though muscle involvement is common, causing symptoms such as weakness, cramps, and myalgia, rhabdomyolysis due to muscle necrosis is uncommon [8]. Only a few cases of rhabdomyolysis have been reported in the literature. The exact pathophysiology is unclear, but the proposed mechanism includes altered oxidative metabolism and the expression of contractile proteins [8]. Other common causes of rhabdomyolysis, such as trauma including crush syndrome and prolonged immobilization, excessive exertion, drugs, infections, metabolic and electrolyte abnormalities, and hyperthermia, should be ruled out [9]. In our case, we ruled out these common causes from history and laboratory analysis. Diagnosis involves marked elevation of CK level (usually less than 10 times the normal when it is due to hypothyroidism as in this case), thyroid function test suggestive of hypothyroidism, and ruling out of other common causes of rhabdomyolysis as discussed above [10,11]. The diagnosis can be confirmed with elevated TPO antibodies. Anti-thyroglobulin antibodies could also be obtained but are less specific [12].

The treatment of rhabdomyolysis includes early and aggressive fluid administration with crystalloid fluids (isotonic saline preferred) at 1-2 L per hour to prevent or treat acute kidney injury with fluid titration based on the patient’s volume status and urine output. Patients with hemolysis need a higher initial rate of 200-300 mL per hour. In addition, patients should be closely followed up with metabolic abnormalities such as hypocalcemia, hyperkalemia, and hyperuricemia [13]. Treatments include the supplementation of synthetic thyroxine (T4, levothyroxine) at approximately 1.6 mcg/kg per dose with a titration dose every 4-6 weeks. Creatine kinase levels usually decline within three to five days of the start of treatment, and patients usually have symptomatic improvement within two weeks [14].

## Conclusions

Even though Hashimoto’s thyroiditis is more common in females and usually presents with mild subclinical symptoms, it can rarely present as overt hypothyroidism, such as rhabdomyolysis in this case. Hence, hypothyroidism should be considered a differential diagnosis of rhabdomyolysis even if there is no prior history of hypothyroidism, especially when the cause is unclear after common causes have been ruled. Early diagnosis and immediate therapy are essential to avoid additional complications.
